# Supplementary material for: Digital health solutions and integrated COVID-19 and TB services to help recover TB care and prevention services in the COVID-19 pandemic: A descriptive study in four high TB burden countries
Source: PLoS One. 2023 Nov 20;18(11):e0293964. doi: 10.1371/journal.pone.0293964 (PMC10659175; doi:10.1371/journal.pone.0293964)
Supplement: S1 Questionnaire — (PDF) [file pone.0293964.s001.pdf]

Supporting Information 1
Landscape assessment

The COVID-19 pandemic has impacted TB services. Worldwide, declines in TB notification rates and worsened TB treatment outcomes are observed. In response, countries have implemented several mitigation strategies. This landscape assessment is part of a project led by KNCV Tuberculosis Foundation and aims to review and better understand the impact of COVID-19 on national TB notification and treatment outcome, and TB and COVID-19 integrated responses. Insights obtained from this landscape assessment will enable us to enhance dissemination and promote the use of guidelines and tools about recent best practices in COVID-19 and TB integrated responses in settings with high TB burden.

PART I: National COVID-19 response

1. Please indicate if COVID-19 restrictions were in place during the (sub)period Q1 2020 till Q1 2021: *
   - ☐ No, there were no COVID-19 restrictions in place
   - ☐ Yes, COVID-19 restrictions were in place

Country	Response
Kyrgyzstan	Yes, COVID-19 restrictions were in place
Nigeria	Yes, COVID-19 restrictions were in place
Tanzania	Yes, COVID-19 restrictions were in place
Vietnam	Yes, COVID-19 restrictions were in place

2. When did these restrictions involve any type of lockdown?
   - ☐ Q1 2020
   - ☐ Q2 2020
   - ☐ Q3 2020
   - ☐ Q4 2020
   - ☐ Q1 2021
   - ☐ Q2 2021
   - ☐ Q3 2021
   - ☐ Q4 2021

Country	Response
Kyrgyzstan	Q2 2020;
Nigeria	Q2 2020;Q3 2020 ;
Tanzania	Q1 2020 ;Q2 2020;Q1 2021 ;
Vietnam	Q2 2020;Q3 2020 ;Q1 2021 ;

3. When did these restrictions involve social distancing (=at least 1-meter personal distance)?
   - ☐ Q1 2020
   - ☐ Q2 2020
   - ☐ Q3 2020
   - ☐ Q4 2020
   - ☐ Q1 2021
   - ☐ Q2 2021
   - ☐ Q3 2021
   - ☐ Q4 2021

Country	Response
Kyrgyzstan	Q2 2020;Q3 2020 ;Q4 2020 ;
Nigeria	Q2 2020;Q3 2020 ;Q1 2020 ;Q4 2020 ;Q1 2021 ;
Tanzania	Q1 2020 ;Q2 2020;Q3 2020 ;Q4 2020 ;Q1 2021 ;
Vietnam	Q2 2020;Q3 2020 ;Q1 2021 ;

4. When did these restrictions involve wearing face masks in public?
   - ☐ Q1 2020
   - ☐ Q2 2020
   - ☐ Q3 2020
   - ☐ Q4 2020
   - ☐ Q1 2021
   - ☐ Q2 2021
   - ☐ Q3 2021
   - ☐ Q4 2021

Country	Response
Kyrgyzstan	Q2 2020;Q3 2020 ;Q4 2020 ;Q1 2021 ;
Nigeria	Q1 2020 ;Q2 2020;Q3 2020 ;Q4 2020 ;Q1 2021 ;
Tanzania	Q1 2020 ;Q2 2020;Q3 2020 ;Q4 2020 ;Q1 2021 ;
Vietnam	Q2 2020;Q3 2020 ;Q1 2021 ;

5. When did these restrictions involve limited availability of public transportation?
   - ☐ Q1 2020
   - ☐ Q2 2020
   - ☐ Q3 2020
   - ☐ Q4 2020
   - ☐ Q1 2021
   - ☐ Q2 2021
   - ☐ Q3 2021
   - ☐ Q4 2021

Country	Response
Kyrgyzstan	Q2 2020;Q3 2020 ;
Nigeria	Q2 2020;Q3 2020 ;
Tanzania	Q1 2020 ;Q2 2020;Q3 2020 ;Q4 2020 ;Q1 2021 ;
Vietnam	Q2 2020;Q3 2020 ;Q1 2021 ;

6. Has your country's national guideline been updated to include TB and COVID-19 integrated testing or screening?
   - ☐ No
   - ☐ Yes, by adding an algorithm
   - ☐ Yes, by adjusting the overall guideline
   - ☐ Do not know

Country	Response
Kyrgyzstan	No
Nigeria	Yes, by adding an algorithm
Tanzania	Yes, by adjusting the overall guideline
Vietnam	Yes, by adding an algorithm

7. Were updated guidelines implemented adequately?
   - ☐ No
   - ☐ Yes

- [ ] Do not know

Country	Response
Kyrgyzstan	Do not know
Nigeria	Do not know
Tanzania	Yes
Vietnam	Do not know

PART II: Operation of TB care services and facilities

8. Have TB notification rates declined since the COVID-19 pandemic?
   - [ ] No
   - [ ] Yes
   - [ ] Do not know

Country	Response
Kyrgyzstan	Yes
Nigeria	No
Tanzania	Yes
Vietnam	No

9. What factors contributed to this decline in TB notification rates?
   - [ ] Fear of patients to seek care
   - [ ] Inability of TB care staff to attend work
   - [ ] Shortages of TB diagnostics
   - [ ] Lockdown
   - [ ] Public transportation restrictions
   - [ ] Other

Country	Response
Kyrgyzstan	Public transportation restrictions; Lockdown; Shortages of TB diagnostics; Inability of TB care staff to attend work; Fear of patients to seek care; Health care facilities focused on COVID-19;
Nigeria	No response
Tanzania	Fear of patients to seek care;
Vietnam	No response

10. Have TB treatment outcomes worsened since the COVID-19 pandemic?
    - [ ] No
    - [ ] Yes
    - [ ] Do not know

Country	Response
Kyrgyzstan	No
Nigeria	Do not know
Tanzania	No
Vietnam	No

11. What factors contributed to the worsening of TB treatment outcomes?
    - ☐ Fear of patients to seek care
    - ☐ Inability of TB care staff to attend work
    - ☐ Shortages of TB diagnostics
    - ☐ Shortages of TB medication
    - ☐ Lockdown
    - ☐ Public transportation restrictions
    - ☐ Other

**Country**	**Response**
Kyrgyzstan	N.A.
Nigeria	N.A.
Tanzania	N.A.
Vietnam	N.A.

12. Please indicate if- and to what extent- active TB case finding/screening has been compromised by the COVID-19 pandemic?
    - ☐ Operate at same level
    - ☐ Operate at lower level
    - ☐ Non- operative

**Country**	**Response**
Kyrgyzstan	Operate at lower level
Nigeria	Operate at lower level
Tanzania	Operate at lower level
Vietnam	Operate at lower level

13. Please indicate if- and to what extent- DS-TB diagnostic services have been compromised by the COVID-19 pandemic?
    - ☐ Operate at same level
    - ☐ Operate at lower level
    - ☐ Non- operative

**Country**	**Response**
Kyrgyzstan	Operate at lower level
Nigeria	Operate at same level
Tanzania	Operate at lower level
Vietnam	Operate at same level

14. Please indicate if- and to what extent- DR-TB diagnostic services have been compromised by the COVID-19 pandemic?
    - ☐ Operate at same level
    - ☐ Operate at lower level
    - ☐ Non- operative

Country	Response
Kyrgyzstan	Operate at lower level
Nigeria	Operate at same level
Tanzania	Operate at lower level
Vietnam	Operate at same level

15. Please indicate if- and to what extent- DS-TB treatment services have been compromised by the COVID-19 pandemic? *
    - ☐ Operate at same level
    - ☐ Operate at lower level
    - ☐ Non- operative

Country	Response
Kyrgyzstan	Operate at lower level
Nigeria	Operate at same level
Tanzania	Operate at lower level
Vietnam	Operate at same level

16. Please indicate if- and to what extent- DR-TB treatment services have been compromised by the COVID-19 pandemic? *
    - ☐ Operate at same level
    - ☐ Operate at lower level
    - ☐ Non- operative

Country	Response
Kyrgyzstan	Operate at lower level
Nigeria	Operate at same level
Tanzania	Operate at lower level
Vietnam	Operate at same level

17. Please indicate if- and to what extent- TB preventive services/treatment have been compromised by the COVID-19 pandemic? *
    - ☐ Operate at same level
    - ☐ Operate at lower level
    - ☐ Non- operative

Country	Response
Kyrgyzstan	Operate at lower level
Nigeria	Operate at same level
Tanzania	Operate at lower level
Vietnam	Operate at same level

18. Have TB healthcare facilities experienced any of the following shortages due to COVID-pandemic?
    - ☐ No, no shortages have occurred due to COVID-19
    - ☐ Yes, TB diagnostics
    - ☐ Yes, DS-TB medication

- ☐ Yes, DR-TB medication
- ☐ Yes, Personal Protective Equipment (PPE)
- ☐ Do not know
- ☐ Other

Country	Response
Kyrgyzstan	Other: HR and facilities - TB facilities health care workers/staff and facilities was involved in responding the COVID-19 measures ;
Nigeria	Yes, DS-TB medication;<br>Yes, DR-TB medication;
Tanzania	No, no shortages have occurred due to COVID-19;<br>Yes, Personal Protective Equipment (PPE);
Vietnam	No, no shortages have occurred due to COVID-19;

19. Please indicate which preventive measures were used by your country to retain TB care services during the COVID-19 pandemic?
    - ☐ No preventive measures were used
    - ☐ Yes, they integrated COVID-19 and TB prevention measures (face masks, hygiene, etc)
    - ☐ Yes, they encouraged TB care staff to continue activities by providing PPE to healthcare facilities
    - ☐ Other

Country	Response
Kyrgyzstan	Yes, they integrated COVID-19 and TB prevention measures (face masks, hygiene, etc);
Nigeria	Yes, they integrated COVID-19 and TB prevention measures (face masks, hygiene, etc);<br>Yes, they encouraged TB care staff to continue activities by providing PPE to healthcare facilities;
Tanzania	Yes, they encouraged TB care staff to continue activities by providing PPE to healthcare facilities;<br>Yes, they integrated COVID-19 and TB prevention measures (face masks, hygiene, etc);
Vietnam	Yes, they integrated COVID-19 and TB prevention measures (face masks, hygiene, etc);

20. Please indicate the strategies used by your country to retain TB screening and diagnostics services during the COVID-19 pandemic?
    - ☐ COVID-19 and TB integrated screening
    - ☐ Home collection and transportation of specimen
    - ☐ Increase in diagnostic capacity of both COVID-19 and TB
    - ☐ Communication of diagnostic outcomes via SMS
    - ☐ Other

Country	Response
Kyrgyzstan	N/A
Nigeria	COVID-19 and TB integrated screening;<br>Increase in diagnostic capacity of both COVID-19 and TB;

	Communication of diagnostic outcomes via SMS;
Tanzania	COVID-19 and TB integrated screening;
Vietnam	COVID-19 and TB integrated screening;<br>Increase in diagnostic capacity of both COVID-19 and TB;<br>Communication of diagnostic outcomes via SMS;

21. Please indicate the strategies used by your country to retain TB treatment services during the COVID-19 pandemic?
    - ☐ Home delivery of medications
    - ☐ Medication refill and monitoring service
    - ☐ Telephone follow-up treatment support
    - ☐ Increase in use of Video Observed Therapy (VOT)
    - ☐ Increase in use of community health workers / volunteers to support treatment
    - ☐ Other

**Country**	**Response**
Kyrgyzstan	Increase in use of Video Observed Therapy (VOT);<br>Increase in use of community health workers / volunteers to support treatment;
Nigeria	Telephone follow-up treatment support;<br>Increase in use of community health workers / volunteers to support treatment;<br>Increase in use of Video Observed Therapy (VOT);<br>There is no NTP report on this question;
Tanzania	Medication refill and monitoring service;
Vietnam	Medication refill and monitoring service;<br>Telephone follow-up treatment support;<br>Increase in use of community health workers / volunteers to support treatment;

22. Please indicate the strategies used by your country to retain TB monitoring & evaluation activities?
    - ☐ Digital monitoring and evaluation meetings
    - ☐ More real time notification and treatment outcome data
    - ☐ Digital notification tools
    - ☐ Other

**Country**	**Response**
Kyrgyzstan	Digital monitoring and evaluation meetings;
Nigeria	Digital monitoring and evaluation meetings;<br>Digital notification tools;
Tanzania	More real time notification and treatment outcome data;
Vietnam	Digital monitoring and evaluation meetings;<br>There is no NTP report on this question;

23. Please indicate the strategies used by your country to retain human resources in TB care services during the COVID-19 pandemic?

- ☐ Virtual training of healthcare workers / community leaders in COVID-19 and TB integrated responses
- ☐ and activities
- ☐ Deployment of lay-providers / non-TB health workers
- ☐ Other

Country	Response
Kyrgyzstan	Virtual training of healthcare workers / community leaders in COVID-19 and TB integrated responses and activities;
Nigeria	Virtual training of healthcare workers / community leaders in COVID-19 and TB integrated responses and activities;<br>Deployment of lay-providers / non-TB health workers;
Tanzania	Use of PPEs in the clinics;
Viet Nam	Virtual training of healthcare workers / community leaders in COVID-19 and TB integrated responses and activities;

PART II: Patient care seeking behavior

24. Please indicate if patient care seeking behavior for TB services has been affected by COVID-19?
    - ☐ No
    - ☐ Yes, patient care seeking for TB symptoms has decreased
    - ☐ Yes, patient care seeking for TB diagnosis has decreased
    - ☐ Yes, patient care seeking for TB treatment has decreased
    - ☐ Do not know
    - ☐ Other

Country	Response
Kyrgyzstan	Yes, patient care seeking for TB symptoms has decreased;<br>Yes, patient care seeking for TB diagnosis has decreased;
Nigeria	Yes, patient care seeking for TB symptoms has decreased;<br>Yes, patient care seeking for TB diagnosis has decreased;
Tanzania	Yes, patient care seeking for TB symptoms has decreased;
Viet Nam	Yes, patient care seeking for TB diagnosis has decreased;<br>Yes, patient care seeking for TB treatment has decreased;<br>There is no data collection/survey available in the country on this question;

25. Please indicate if -and how- the location of care seeking has changed by COVID-19?
    - ☐ No, location of seeking care has not changed at all
    - ☐ Yes, patients seek more care at informal healthcare facilities (pharmacy, traditional healers)
    - ☐ Yes, patients seek more care at primary care levels (such as local public or private health centers)
    - ☐ Yes, patients now seek more care at secondary levels (such as hospitals)
    - ☐ Do not know
    - ☐ Other

Country	Response

Kyrgyzstan	Yes, patients seek more care at primary care levels (such as local public or private health centers);
Nigeria	Initially at the peak of COVID-19 patients sought care mainly at informal and private health facilities but as COVID-19 restrictions eased patients gradually returned to original care seeking behaviour. ;
Tanzania	Yes, patients seek more care at primary care levels (such as local public or private health centers);
Viet Nam	Do not know;<br>There is no data collection/survey available in the country on this question;

26. Please indicate if and how TB treatment support has changed since COVID-19?
    - ☐ No
    - ☐ Yes, patients are increasingly taking medication under own observation
    - ☐ Yes, patients are increasingly taking medication under direct digital observation (for example VOT)
    - ☐ Yes, patients are increasingly taking medication under direct observation by community workers
    - ☐ Do not know
    - ☐ Other

Country	Response
Kyrgyzstan	Yes, patients are increasingly taking medication under direct digital observation (for example VOT);
Nigeria	Yes, patients are increasingly taking medication under direct observation by community workers;
Tanzania	Yes, patients are increasingly taking medication under own observation;
Viet Nam	Do not know;<br>There is no data collection/survey available in the country on this question;

PART III: Tuberculosis (TB) care staff

27. Were human resources involved in TB care and prevention activities affected by COVID-19?
    - ☐ No, there was no change in the number of healthcare workers
    - ☐ Yes, because of increased sick leave among health care workers
    - ☐ Yes, because TB care staff refrained from work out of fear of COVID-19 infection
    - ☐ Yes, because TB care staff were unable to attend work due to lock down or travel restrictions
    - ☐ Yes, because TB care staff were redeployed in COVID-19 activities
    - ☐ Do not know
    - ☐ Other

Country	Response
Kyrgyzstan	Yes, because of increased sick leave among health care workers;<br>Yes, because TB care staff were redeployed in COVID-19 activities;
Nigeria	Yes, because TB care staff refrained from work out of fear of COVID-19 infection;<br>Yes, because TB care staff were unable to attend work due to lock down or travel restrictions;<br>Yes, because TB care staff were redeployed in COVID-19 activities;
Tanzania	Yes, because TB care staff were redeployed in COVID-19 activities;

Viet Nam	Yes, because TB care staff were redeployed in COVID-19 activities;<br>There is no data collection/survey available in the country on this question;
