# Supplementary material for: Digital health solutions and integrated COVID-19 and TB services to help recover TB care and prevention services in the COVID-19 pandemic: A descriptive study in four high TB burden countries
Source: PLoS One. 2023 Nov 20;18(11):e0293964. doi: 10.1371/journal.pone.0293964 (PMC10659175; doi:10.1371/journal.pone.0293964)
Supplement: S1 File — (PDF) [file pone.0293964.s002.pdf]

Supporting information 2
Country profiles

1. Kyrgyzstan

COVID-19 situation

On the 18th of March 2020, the first COVID-19 patient was notified in Kyrgyzstan. An increase in COVID-19 patients followed. After a peak in the third quarter of 2020 (41163 notified COVID-19 patients), a decline was observed until the first quarter of 2021. Similarly, an increasing trend until the third quarter of 2021 was observed (50731 notified COVID-19 patients) followed by a decline in the third quarter (Figure 1). Following the emergence of COVID-19 in Kyrgyzstan, prevention measures such as lockdown (quarter 2 2020), social distancing, wearing face masks, limited availability of public transportation (quarter 2 and 3 in 2020) were in place from the 2nd quarter 2020 onwards (Table 1).

Figure 1. Trend in notified tuberculosis and COVID-19 patients

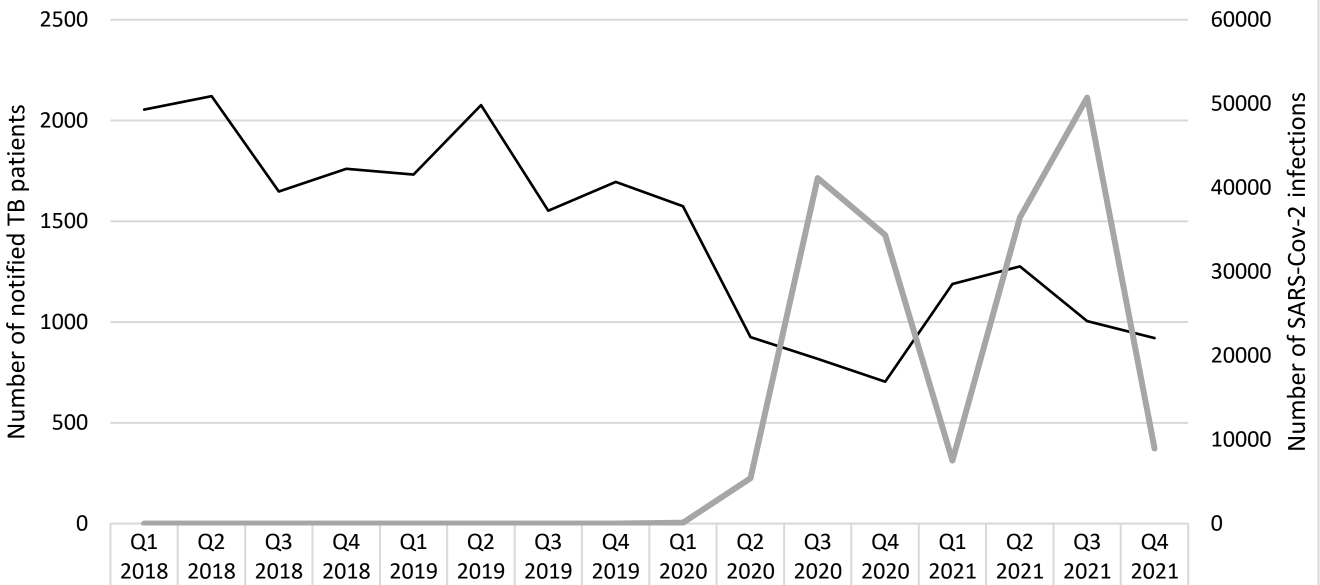

	Q1 2018	Q2 2018	Q3 2018	Q4 2018	Q1 2019	Q2 2019	Q3 2019	Q4 2019	Q1 2020	Q2 2020	Q3 2020	Q4 2020	Q1 2021	Q2 2021	Q3 2021	Q4 2021
Tuberculosis	2055	2121	1648	1761	1732	2076	1553	1696	1575	925	818	704	1189	1276	1004	921
SARS-Cov-2	0	0	0	0	0	0	0	0	107	5399	41163	34365	7504	36465	50731	8955

*Data source TB notification data: https://www.who.int/teams/global-tuberculosis-programme/data and National Tuberculosis Programs
*Data source SARS-Cov-2 infection notification data: Ritchie H, Mathieu E, Rodés-Guirao L, Appel C, Giattino C, Ortiz-Ospina E, et al. Coronavirus Pandemic (COVID-19). Published online at OurWorldInData.org. 2020 [Available from: https://ourworldindata.org/coronavirus.

Table 1. COVID-19 prevention measures in place in the period Q1 2020 – Q1 2021

**Type of COVID-19 prevention measure**	**Period**
Any type of lockdown	Q2 2020
Social distancing (≥1-meter personal distance)	Q2 2020-Q4 2021
Wearing face masks in public	Q2 2020-Q4 2021
Limited availability of public transportation	Q2 2020; Q3 2020

Influence of COVID-19 pandemic on TB notification and services

Following the start of the COVID-19 pandemic, the number of notified tuberculosis (TB) patients has shown a considerable decline (Figure 1). In the first quarter of 2020, the number of notified TB patients dropped from 1696 in quarter 4 2019 to 1575 and 925 in quarter one and two of 2020, respectively. The TB notification trend follows an opposite trend when compared to the COVID-19 notifications: following a decline in the COVID-19 notifications in quarter 1 of 2020, the TB notification trend shows an increase from 704 to 1189 notified TB patients in Q4 2020 and Q1 2021, respectively.

Following the outcomes of the landscape assessment, multiple factors likely contributed to this decline in TB notifications. First, COVID-19 prevention measures such as the lockdown and limited public transportation likely contributed to the decline in notified TB patients. Additionally, shortages of TB diagnostics and TB care staff, and the focus of healthcare facilities on COVID-19 also likely contributed to the decline in TB notifications. The TB care staff capacity has declined because of the inability of staff to attend work, due to sick leave, and due to redeployment of staff to COVID-19 activities.

Finally, as a result of fear, a decline in symptom and diagnostic care seeking behavior was reported, which is likely to also contribute to the decline in TB notifications. Additionally, persons with care needs are reported to have the tendency to seek more care at primary care levels such as local public or private health centers.

Due to the COVID-19 pandemic, all TB services were reported to operate at a lower level compared to pre-pandemic operational levels (Table 2).

Table 2. Level of operations of TB services during the COVID-19 pandemic

Type of TB service	Operational level
Active TB case finding/screening	Operate at lower level
DS-TB diagnostic services	Operate at lower level
DR-TB diagnostic services	Operate at lower level
DS-TB treatment services	Operate at lower level
DR-TB treatment services	Operate at lower level
TB preventive services/treatment	Operate at lower level

Multiple strategies were employed to retain the TB services as much as possible. Firstly, COVID-19 and TB prevention measures (face masks, hygiene, etc) were integrated. Additionally, there has been an increase in the use of direct observed treatment through the employment of video observed therapy and community health workers to support TB treatment. Furthermore, digital monitoring and evaluation meetings were organized, and virtual trainings of healthcare workers and community leaders were developed and organized COVID-19 and TB integrated responses and activities.

Table 3. Strategies implemented to retain TB services

Type of TB service	Strategies for retainment of service
Screening and diagnostics	N/A
Treatment	• Increase in use of Video Observed Therapy (VOT)<br>• Increase in use of community health workers / volunteers to support treatment
Monitor & evaluation	• Digital monitoring and evaluation meetings
Human resources in TB care	• Virtual training of healthcare workers / community leaders in COVID-19 and TB integrated responses and activities

2. Nigeria

COVID-19 situation

On the 28th of March, the first COVID-19 patient was notified in Nigeria. Following this first notification, the trend in COVID-19 patients reached its peak in Q1 2021, after which a large decline in Q2 2021 was followed by an increase in notified COVID-19 patients in Q3 2021 (Figure 1). Following the first notified COVID-19 patients in Q1 2020 and onwards, wearing face masks was implemented as prevention measures followed by social distancing in Q2 2020 and onwards. In addition to these measures, a lockdown and limited availability of public transportation were in place in Q2 and Q3 of 2020.

Figure 1. Trend in notified tuberculosis patients and SARS-Cov-2 infections

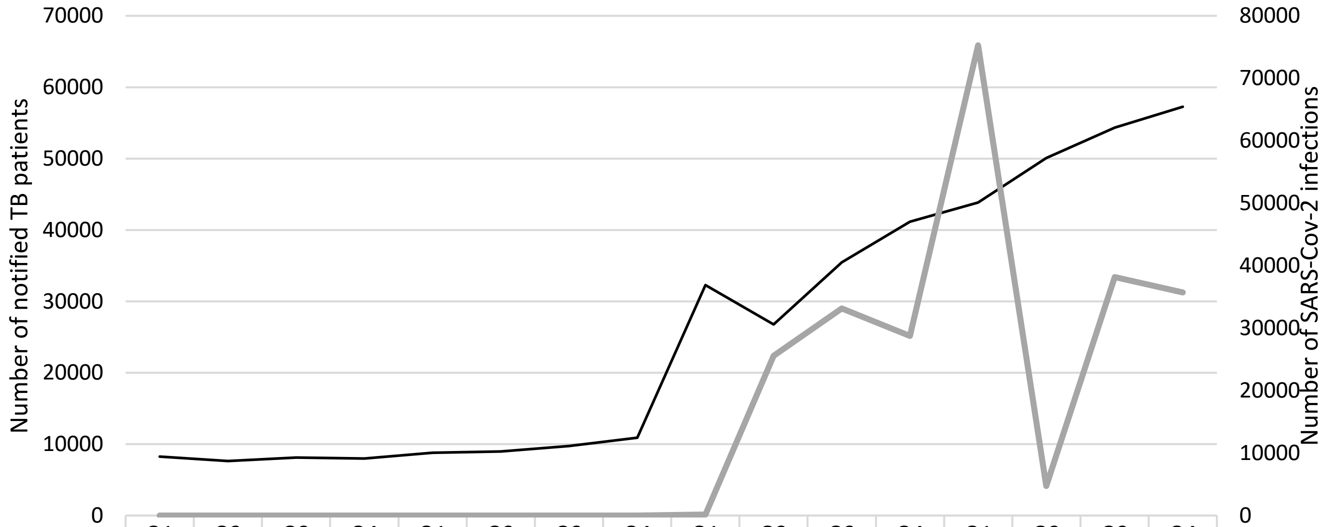

Period	Q1 2018	Q2 2018	Q3 2018	Q4 2018	Q1 2019	Q2 2019	Q3 2019	Q4 2019	Q1 2020	Q2 2020	Q3 2020	Q4 2020	Q1 2021	Q2 2021	Q3 2021	Q4 2021
Tuberculosis	8248	7632	8101	7963	8800	8977	9755	10905	32289	26755	35444	41150	43838	50096	54358	57260
SARS-Cov-2	0	0	0	0	0	0	0	0	135	25559	33154	28759	75284	4727	38161	35734

Period

*Data source TB notification data: https://www.who.int/teams/global-tuberculosis-programme/data and National Tuberculosis Programs
*Data source SARS-Cov-2 infection notification data: Ritchie H, Mathieu E, Rodés-Guirao L, Appel C, Giattino C, Ortiz-Ospina E, et al. Coronavirus Pandemic (COVID-19). Published online at OurWorldInData.org. 2020 [Available from: https://ourworldindata.org/coronavirus.

Table 1. COVID-19 prevention measures in place in a given period

Type of COVID-19 prevention measure	Period
Any type of lockdown	Q2 2020; Q3 2020;
Social distancing (≥1-meter personal distance)	Q1 2020-Q4 2021;
Wearing face masks in public	Q1 2020-Q4 2021;
Limited availability of public transportation	Q2 2020; Q3 2020;

Influence of COVID-19 pandemic on TB notification and services

The trend in notified TB patients only showed a small decline between Q1 2020 and Q2 2020 following the emergence of COVID-19 in Nigeria. After Q2 2020, the trend in notified TB patients has increased ever since. (Figure 1) During the first peak in notified COVID-19 patients, healthcare seeking behavior was reported to be decreased and shifted to informal and private health facilities.

Regarding the TB prevention and care services, only services on active TB case finding and screening have been operating at a lower level. Other services were retained on same pre-pandemic operation levels. However, TB care

facilities have experienced shortages in medication for both drug-susceptible and drug-resistant TB. Furthermore, TB human resources were compromised because TB care staff refrained from work out of fear of COVID-19 infection, were unable to attend work due to lock down or travel restrictions, and/or were redeployed in COVID-19 activities. (Table 2)

Table 2. Level of operations of TB services during the COVID-19 pandemic

Type of TB service	Operational level
Active TB case finding/screening	Operate at lower level
DS-TB diagnostic services	Operate at same level
DR-TB diagnostic services	Operate at same level
DS-TB treatment services	Operate at same level
DR-TB treatment services	Operate at same level
TB preventive services/treatment	Operate at same level

Other measures employed to retain TB care and prevention services are integrated COVID-19 and prevention measures (such as; face masks, hygiene, etc). Furthermore, healthcare facilities are provided with appropriate PPE to encourage their staff to continue activities. Digital health strategies are employed to retain multiple TB services. For example, communication of diagnostic outcomes is done via text messaging, treatment support is provided through telephone, and digital meetings are organized to e4xecute monitoring and evaluation and training of healthcare workers.

Furthermore, non-TB health workers are employed to support and execute local TB care and prevention services. For example, patients are increasingly taking medication under direct observation by community workers.

Table 3. Strategies implemented to retain TB services

Type of TB service	Strategies for retainment of service
Screening and diagnostics	• COVID-19 and TB integrated screening<br>• Increase in diagnostic capacity of both COVID-19 and TB<br>• Communication of diagnostic outcomes via SMS;
Treatment	• Medication refill and monitoring service<br>• Telephone follow-up treatment support<br>• Increase in use of community health workers / volunteers to support treatment
Monitor & evaluation	• Digital monitoring and evaluation meetings<br>• Digital notification tools
Human resources in TB care	• Virtual training of healthcare workers / community leaders in COVID-19 and TB integrated responses and activities<br>• Deployment of lay-providers / non-TB health workers

TB and COVID-19 integrated responses

The national guideline has been updated by adding an algorithm to include TB and COVID-19 integrated testing or screening. However, it is unknown whether these guidelines were implemented adequately. Screening for COVID-19 and TB are integrated.

3. Tanzania

COVID-19 situation

On 16 March 2020, the first COVID-19 patient was notified in Tanzania followed by a number of notified COVID-19 patients in 2020 (total of 509). The notification of COVID-19 patients in Tanzania shows an irregular trend with 24307 notified patients on 23rd of September 2021, followed by a decline in Q4 2021. (Figure 1) Following the emergence of COVID-19 in March 2020 in Tanzania, no COVID-19 restrictions have been in place between Q1 2020 and Q1 2021. (Table 1)

Figure 1. Trend in notified tuberculosis patients and SARS-Cov-2 infections

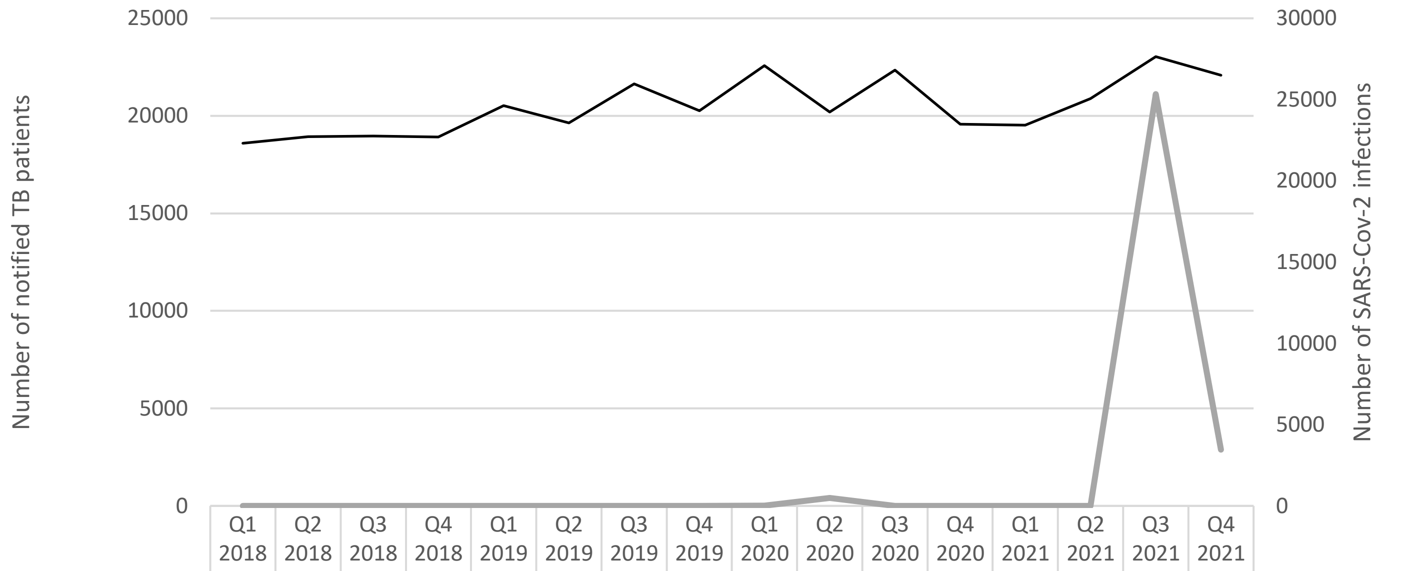

Period	Q1 2018	Q2 2018	Q3 2018	Q4 2018	Q1 2019	Q2 2019	Q3 2019	Q4 2019	Q1 2020	Q2 2020	Q3 2020	Q4 2020	Q1 2021	Q2 2021	Q3 2021	Q4 2021
Tuberculosis	18593	18931	18972	18913	20529	19630	21636	20263	22581	20193	22347	19565	19522	20881	23034	22089
SARS-Cov-2	0	0	0	0	0	0	0	0	19	490	0	0	0	0	25337	3460

*Data source TB notification data: https://www.who.int/teams/global-tuberculosis-programme/data and National Tuberculosis Programs
*Data source SARS-Cov-2 infection notification data: Ritchie H, Mathieu E, Rodés-Guirao L, Appel C, Giattino C, Ortiz-Ospina E, et al. Coronavirus Pandemic (COVID-19). Published online at OurWorldInData.org. 2020 [Available from: https://ourworldindata.org/coronavirus.

Table 1. COVID-19 prevention measures in place in a given period

Type of COVID-19 prevention measure	Period
Any type of lockdown	None
Social distancing (≥1-meter personal distance)	None
Wearing face masks in public	Q2 2021 – Q4 2021
Limited availability of public transportation	None

Influence of COVID-19 pandemic on TB notification and services

Since the emergence of COVID-19 in Tanzania, the TB notification trend showed a small decline between Q1 2020 and Q4 2020, after which the number of notified TB patients has increased to pre-pandemic levels. The decline in TB notifications could potentially be explained by a reported decline in patients care seeking behavior for symptoms or diagnostics because of fear for COVID-19. Additionally, healthcare seeking behavior was reported to be shifted towards more informal healthcare facilities such as pharmacies and traditional healers. Following the COVID-19 emergence in Tanzania, only the active case finding and screening for TB services have been operating at a lower level. Other services

have been operating at similar levels as pre-pandemic. It is unknown if any shortages in diagnostics or medications were experienced. (Table 2)

Table 2. Level of operations of TB services during the COVID-19 pandemic

Type of TB service	Operational level
Active TB case finding/screening	Operate at lower level
DS-TB diagnostic services	Operate at same level
DR-TB diagnostic services	Operate at same level
DS-TB treatment services	Operate at same level
DR-TB treatment services	Operate at same level
TB preventive services/treatment	Operate at same level

Multiple strategies were implemented to retain TB care and preventions services. The main strategies involve limiting the contact moments with healthcare workers. For example, specimen collection for diagnostics is done more at home and outcomes are communicated through text messaging. Additionally, TB treatment is increasingly taken under patients' own observations and follow-up and support is provided through phone calls.

Table 3. Strategies implemented to retain TB services

Type of TB service	Strategies for retainment of service
Screening and diagnostics	• Communication of diagnostic outcomes via SMS<br>• Home collection and transportation of specimen
Treatment	• Telephone follow-up treatment support<br>• Increase in use of community health workers / volunteers to support treatment
Monitor & evaluation	• More real time notification and treatment outcome data
Human resources in TB care	• No particular strategy deployed

TB and COVID-19 integrated responses

There was no update of the national guideline to include TB and COVID-19 integrated testing or screening. However, integrated COVID-19 and TB prevention measures are in place (such as face masks, hygiene, etc).

4. Vietnam

COVID-19 situation

On the 23rd of January 2020, the first patient with COVID-19 was notified in the data system. Following a small increase in notified patients in the following year, a large increase was observed in Q2 2021. Following the COVID-19 emergence, Vietnam has implemented multiple prevention measures in Q2 2020 until Q2021, such as lock down, social distancing, wearing face masks, and limited availability of public transportation.

Figure 1. Trend in notified tuberculosis patients and SARS-Cov-2 infections

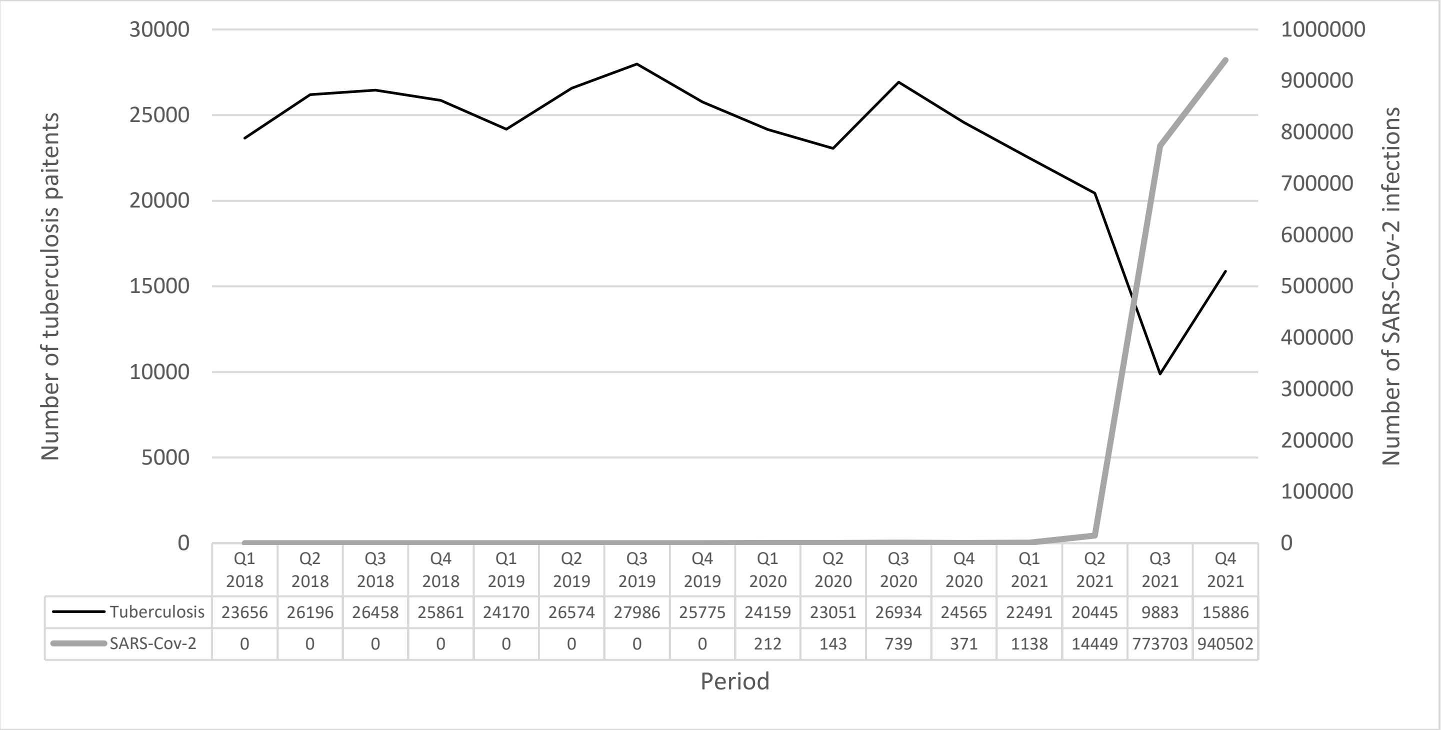

*TB notification data for the period Q1 2018-Q4 2019 is average of annual TB notification data; quarterly data on TB treatment uptake and completion not available
*Data source TB notification data: https://www.who.int/teams/global-tuberculosis-programme/data and National Tuberculosis Programs
*Data source SARS-Cov-2 infection notification data: Ritchie H, Mathieu E, Rodés-Guirao L, Appel C, Giattino C, Ortiz-Ospina E, et al. Coronavirus Pandemic (COVID-19). Published online at OurWorldInData.org. 2020 [Available from: https://ourworldindata.org/coronavirus.

Table 1. COVID-19 prevention measures in place in the period Q1 2020 – Q1 2021

Type of COVID-19 prevention measure	Period
Any type of lockdown*	Q2 2020-Q4 2021
Social distancing (≥1-meter personal distance)	Q2 2020-Q4 2021
Wearing face masks in public	Q2 2020-Q4 2021
Limited availability of public transportation	Q2 2020-Q4 2021

*Under directives 15 (stop gathering events with more than 20 people per room; stop all cultural activities, sports, entertainment activities; do not gather >10 people outside of offices, hospitals, and schools) and 16 (Social isolation: everyone must stay home, only going out when absolutely necessary; do not gather more than 2 people outside of offices, hospitals, schools, and public spaces).

Influence of COVID-19 pandemic on TB notification and services

The trend in notified TB patients shows a small decline between Q4 2019 and Q2 2021. The TB care and prevention services have been reported to function at pre-pandemic levels, except for active TB case finding and screening. This may explain the small decline in TB notifications. Another explanation might be the reported decline in patient care

seeking behavior. (Table 2) No shortages in TB diagnostics or medications have been reported. Additionally, TB human resources have been compromised due to the redeployment of TB care staff in COVID-19 activities.

Table 2. Level of operations of TB services during the COVID-19 pandemic

Type of TB service	Operational level
Active TB case finding/screening	Operate at lower level
DS-TB diagnostic services	Operate at same level
DR-TB diagnostic services	Operate at same level
DS-TB treatment services	Operate at same level
DR-TB treatment services	Operate at same level
TB preventive services/treatment	Operate at same level

Multiple strategies have been employed by Vietnam in retaining TB care and prevention services. For example, by integrating and increasing the capacity of integrated COVID-19 and TB screening services. Additionally, digital health strategies have been implemented such as the communication of diagnostic outcomes through text messaging, follow-up treatment support through phone calls and video observed therapy, and digital meetings for monitoring and evaluation activities and capacity building. Other strategies are listed in Table 3.

Table 3. Strategies implemented to retain TB services

Type of TB service	Strategies for retainment of service
Screening and diagnostics	• COVID-19 and TB integrated screening<br>• Increase in diagnostic capacity of both COVID-19 and TB<br>• Communication of diagnostic outcomes via SMS
Treatment	• Telephone follow-up treatment support<br>• Increase in use of community health workers / volunteers to support treatment<br>• Increase in use of Video Observed Therapy (VOT)
Monitor & evaluation	• Digital monitoring and evaluation meetings<br>• There is no NTP report on this question;
Human resources in TB care	• Virtual training of healthcare workers / community leaders in COVID-19 and TB integrated responses and activities;

TB and COVID-19 integrated responses

The country's national guideline has been updated to include TB and COVID-19 integrated testing or screening by adding an algorithm. It is unknown whether this updated guideline has been implemented adequately. Yes, they integrated COVID-19 and TB prevention measures (face masks, hygiene, etc).
