# Supplementary material for: Digital health solutions and integrated COVID-19 and TB services to help recover TB care and prevention services in the COVID-19 pandemic: A descriptive study in four high TB burden countries
Source: PLoS One. 2023 Nov 20;18(11):e0293964. doi: 10.1371/journal.pone.0293964 (PMC10659175; doi:10.1371/journal.pone.0293964)
Supplement: Attachment — Submitted filename: Response to reviewers (1).pdf [file pone.0293964.s004.pdf]

Manuscript title: Digital health solutions and integrated COVID-19 and TB services to help recover TB care and prevention services in the COVID-19 pandemic: a descriptive study in four high TB burden countries

Manuscript ID: PONE-D-22-12851

April 13, 2023, The Hague, The Netherlands

Dear editor,

Thank you for the opportunity to submit a revised version of our manuscript. We are grateful for the reviewers' comments, which we believe were helpful to strengthen our paper. We responded to the best of our capabilities. We responded to the comments one-by-one below in the section 'Response to the Reviewers'. Following the journal requirements, we have updated the following references to meet the Vancouver style. We noticed that reference number 1 and 9 were the same (REF: Global Tuberculosis Report 2021. Geneva: World Health Organization; 2021:Licence: CC BY-NC-SA 3.0 IGO.). We removed number 9 and ensured that number 1 was inserted instead.

Furthermore, we updated our collected data and consequent analysis to include the periods Q2 2021, Q3 2021, and Q4 2021. We were able to retrieve quarterly TB notification data for Vietnam, which enabled us to include Vietnam in the statistical analysis.

We look forward to your decision regarding acceptance of the revised manuscript.

On behalf of the authors,
Kind regards

Ineke Spruijt
TB consultant and epidemiologist

KNCV TUBERCULOSEFONDS

Postbus 146,
2501 CC The Hague

Visiting adress: Maanweg 174, 2516 AB, The Hague, The Netherlands

---

Response to the reviewers

Reviewer #1: I think the manuscript has improved compared to the previous version, but there are points that still need clarification/revision as detailed below.
1. Line 18: perhaps this should read "declined by 18%"? Corrected
2. Line 69: what is transmitted is the pathogen, not the disease – please amend. Amended
3. Lines 104-105: I suppose that "wearing face masks in public spaces" refers to whether a mandatory masking policy was in place. If so, I would suggest rephrasing for the sake of clarity. Amended
4. Line 105: please provide a definition of "limited availability of public transportation". I think this is too vague as stated. We added: (i.e. reduced availability of public transportation compared to pre-COVID pandemic)

5. Lines 107: what are “MS forms”? Please provide the full name of the platform along with its developer. Adjusted into “Microsoft Forms”.
6. Lines 99-108: from this paragraph it remains unclear to me whether the questionnaire was filled out by country project officers or by others who were contacted by project officers as part of the landscape assessment. Adjusted into: The landscape assessment consisted of an online questionnaire filled in by the country representatives which we designed in Microsoft Forms.
7. Lines 118-119 and subsequent text: please make sure that the WHO’s Global TB Programme is correctly mentioned (it shouldn’t be “Global TB Program”). Adjusted into: “the WHO’s Global TB Programme”
8. Line 136: it is unclear to me why only univariable models were used to evaluate the association between a range of IPC measures and TB notifications. Given that more than one measure was typically implemented in a given country, I would expect the effect of these concomitant measures to sum up, thus becoming hard to disentangle. I believe that the resulting crude associations overestimate the impact of individual approaches. Please explain the rationale for doing so and consider undertaking multivariable analyses as well.
We agree with this comment and should have combined the measures taken during the same quarterly periods (as they yield the same changes in TB notifications during that period and the effect of an individual measure on the notifications cannot be calculated). The measures taken during different quarterly periods should be included in a multivariable model. When adjusting the analysis, we only could employ a multivariable model for Kyrgyzstan. The results of adjusted analysis are presented in the manuscript. [Page 13-14, Lines 253-263 & Table 5] We also adjusted the methods section to accommodate changes made to the analysis. [Page 7-8, Lines 151-161]
9. Lines 145-146: I am aware that the definition of “overdispersion” is that the “variance exceeds the mean”. However, this could be made clearer in the text, especially for readers who are less familiar with these concepts. For instance, I would suggest clarifying the variance and mean of which variable(s) you are referring to. A bit more of elaboration would help.
We have adjusted the following sentence: “During exploratory analysis, we detected overdispersion in the notification data: **the variance of the response variable (TB notifications) exceeded that of its mean.**” [Page 7, Lines 152-154]
10. Lines 167-169: this sentence is confusing – please rephrase. Also, while providing absolute numbers is fine and meaningful, I would add percent increases relative to the number of tests done. Absolute numbers alone may not be fully reflective of the actual epidemic trends. Similar considerations apply to the next few sentences that report findings in the same format.
Thank you for your suggestion. We adjusted the sentences into the following: *“Following the emergence of the COVID-19 pandemic in Q1 2020, the number of notified SARS-CoV-2 infections increased most rapidly from 5399 to 41163 infections (662%)) between Q2 2020-Q3 2020 in Kyrgyzstan; from 28759 to 75284 infections (162%) between Q4 2020-Q1 2021 in Nigeria; and from 14449 to 773703 infections (5255% increase) between Q2 2021 and Q3 2021) in Vietnam. . The increase in SARS-CoV-2 infections in Tanzania started after Q2 2021 (from 0 to 25337 infections) (Fig. 1).”* [Page 8, Lines 176-184]
11. Table 2 is not completely clear. I think it is important to make the term of comparison explicit. This is especially important considering that tables and figures should stand-alone and thus contain all the details needed for interpretation. In this regard, the meaning of “Lower” and “Same” is not straightforward. The table title and notes should be revised accordingly.
We adjusted the table title into the following: *“Table 2. Operational level strategy of tuberculosis diagnostic and treatment services during the pandemic and compared to the pre-COVID-19 pandemic situation per country.”* We adjusted the text in the first row of the table into the following: *“Operational level strategy[1] compared to the pre-COVID-19 pandemic situation per country”*
12. Please make sure that all acronyms are spelled out at their first appearance in the text (e.g. I am not sure that HCW was defined). Done
13. Line 212: communicated to whom? Adjusted into “by HCWs to patients”

14. Lines 227-229: please clarify what this algorithm entails. Is it based on the use of the GeneXpert platform for the simultaneous detection of TB and SARS-CoV-2 on the same specimen or what?
We recognize that the words “screening algorithm” may imply that one specimen is used for testing for both TB and COVID-19. However, in Nigeria and Vietnam, it should be seen more as integration of two screening approaches: one for TB (CXR and GeneXpert) and one for COVID-19 (antigen tests). WE have adjusted the text into the following: *“In Nigeria and Viet Nam, COVID-19 and TB responses were integrated by adopting an integrated COVID-19/TB screening for “emergency use approval”. The screening approach, which consists of TB screening using chest X-ray and confirmatory Gene Xpert testing and COVID-19 screening using antigen testing,”*
15. Line 238: “Statistical analysis” looks like an odd heading for a subsection of the results. For consistency, please replace with something that clearly identifies the content of the paragraph as done with previous subsection. We agree that “Statistical analysis” is not appropriate in the results section. We have changed the heading of this subsection into: *“The association between COVID-19 IPC measures and TB notifications”*
16. Line 239: what does “the installation wearing face masks in public” mean? It refers to the installation of policies. We have adjusted the text into: *“the installation of policies for mandatory masking in public and social distancing (≥1 meter), and limited availability of public transportation was significantly”*
17. Line 299: this analysis does not assess the “effect” of X on Y, but the “association” between X and Y. This is a very important point, even more so given that only crude estimates are reported. Please avoid the use of causal language that is clearly not supported by the study design and approaches. Apologies for the inaccurate use of language. We have adjusted the sentence into the following: *“Our study was only able to statistically assess the association between COVID-19 restrictions and changes in TB notifications in two out of four countries.”*
18. There are a few typos and missing words throughout the manuscript. Please review for spelling/grammar. Thank you for pointing out the typos and missing words. A native English speaker has reviewed the manuscript and corrected any errors.

Reviewer #2: Thanks to the authors for the thorough response to my comments (and those of other reviewers). I think the manuscript's conclusions are now more reflective of the findings and analyses. It's a much smoother manuscript now. The revisions to the methods section help a lot. I am still a bit doubtful about the regression analysis, as I think the qualitative information is more informative and holds more descriptive value. Some further copy-editing would be welcome. Good job
Thank you for your compliments and efforts to review our manuscript. The manuscript was once more reviewed by a native English speaker to do some final copy-editing.

Reviewer #3: the writing is much clearer in the revised version - the manuscript is more focused and flows much better. The methods section in particular is much clearer, and analysis as presented more consistent with the objectives of the study. Fig 1 graphics are also much easier to interpret now. It's a very useful and interesting read!
Thank you for your compliments and efforts to review our manuscript.

There are a few small copyedit errors remaining:
Table 1 Kyrgystan, under Lockdown: "to minors unaccompanied" should be "for minors unaccompanied"
Table 1 Viet Nam, under Lockdown: "onmly" should be "only"
line 146: there is an extra period
line 279: "contributed" should be "attributed"
Thank you for pointing out the typos and errors. We have adjusted the text accordingly. We also asked a native English speaker to review the manuscript and corrected any further errors.

Reviewer #4: This paper has considerable potential to bring this issue into the mainstream through PLOS. I read the study with interest. The authors have already revised and addressed several critical issues raised by other esteemed reviewers. The findings of this study, as revised in Revision -1, meet the standards and would be valuable to the discipline of infectious diseases.
Thank you for your compliments and efforts to review our manuscript.

Reviewer #5: Authors have conducted a retrospective study in their manuscript titled "Digital health solutions and integrated COVID-19 and TB services to help recover TB care and prevention services in the COVID-19 pandemic: a descriptive study in four high TB burden countries". The topic is of utmost importance and pandemic did posed unique challenges to identify and treat TB patients throughout the world. Introduction is well scripted and does intrigue the readers about the importance of topic and the underlying rationale of the study is well justified. Methods are well defined and authors have included most of the of the recommendations of reviewers in their revised manuscripts. Results are detailed and well explained with the help of tables which will be essential for readers to understand. Another way of presenting the results could be dividing the result section based on individual country, but I would leave this consideration to authors discretion. As even in current format they are very intelligibly presented. Limitations are included and are just. Minor spell checking and proof reading is required, especially the spelling of "Vietnam".
Thank you for your compliments and efforts to review our manuscript. We have presented individual country results in the supplements. We also asked a native English speaker to review the manuscript and corrected any further errors.

Reviewer #6: (No Response)
